# Liver Fat, Hepatic Enzymes, Alkaline Phosphatase and the Risk of Incident Type 2 Diabetes: A Prospective Study of 132,377 Adults

**DOI:** 10.1038/s41598-017-04631-7

**Published:** 2017-07-05

**Authors:** Sean Chun-Chang Chen, Shan Pou Tsai, Jing-Yun Jhao, Wun-Kai Jiang, Chwen Keng Tsao, Ly-Yun Chang

**Affiliations:** 1 Graduate Institute of Biomedical Informatics, College of Medical Science and Technology, Taipei Medical University, Taipei, 110 Taiwan; 2 MJ Health Management Institution, Taipei, 114 Taiwan; 3 MJ Health Research Foundation, Taipei, 114 Taiwan; 4 Institute of Sociology, Academia Sinica, Taipei, 115 Taiwan

## Abstract

Previous studies have reported inconsistent results of the associations of alanine transaminase (ALT), aspartate transaminase (AST), gamma-glutamyltransferase (GGT) and alkaline phosphatase (ALP) with incident type 2 diabetes (diabetes hereafter). We aimed to resolve the controversy by taking nonalcoholic fatty liver disease (NAFLD) into account. The study population comprised 132,377 non-diabetic individuals (64,875 men and 67,502 women) aged 35–79 who had two or more health examinations during 1996–2014. A total of 6,555 incident diabetes (3,734 men and 2,821 women) were identified, on average, over 5.8 years of follow-up. Cox regression was used to calculate the hazard ratio (HR) for incident diabetes, adjusting for classical confounders. The risk of incident diabetes was significantly associated with NAFLD [HR = 2.08 (men) and 2.65 (women)]. Elevated ALT, AST, GGT and ALP were also significantly associated with the increased risk of diabetes, with HRs of 1.27, 1.23, 1.58 and 1.37, respectively, in men, and 1.56, 1.18, 1.48 and 1.44, respectively in women. Our results suggest that NAFLD, ALT, AST, GGT and ALP are independent predictors for incident diabetes in both men and women.

## Introduction

The prevalence and incidence of type 2 diabetes (diabetes hereafter) are rising rapidly worldwide, especially in Asia^1^. Diabetes has been linked to a shorter life expectancy mainly because of its complications, including heart disease, strokes, eye disease, kidney failure and bone disease^2^. Lifestyle changes and medications are shown to reduce the incidence of diabetes^3^. Therefore, identification and evaluation of risk factors for diabetes is crucial for early intervention and treatment.

The relationships between hepatic enzymes including alanine transaminase (ALT), aspartate transaminase (AST) and gamma-glutamyltransferase (GGT) and incident diabetes have been examined, but the results are inconsistent. Several prospective studies have reported that ALT was assocaited with incident diabetes^4–9^, but this association was not significant in a study of 4,201 French men and women^10^. Hanley *et al*.^5^, Fraser *et al*.^4^, and Schneider *et al*.^11^ found that AST independently predicted incident diabetes in 906 non-Hispanic Americans, 3,041 British women, and 9,337 Americans (7,495 white and 1,842 black), respectively. However, others showed that AST did not predict incident diabetes^7, 8, 10^. In a recent review of literature, Kunutsor and colleagues^12^ suggested that AST was associated with the risk of incident diabetes after controlling for potential confounding factors. In addition, while most of the studies showed that elevated GGT level was a strong indicator for the onset of diabetes independent of common diabetes risk factors^9, 10^, a study in Pima Indians found GGT not a predictor^8^. Several recent studies used the Mendelian randomization to estimate the causal effects of liver enzymes on incident diabetes. ALT was shown to increase the risk of diabetes^13^ whereas the causal role of GGT in the development of diabetes remained controversial^13–15^.

Non-alcoholic fatty liver disease (NAFLD), the accumulation of excess fat in liver cells that is not caused by alcohol, has been found to be associated with several features of insulin resistance^16^ and incident diabetes^4, 17^. Ultrasonography-diagnosed NAFLD is an independent risk factor for diabetes^18–24^. Notably, most of them were conducted in Japanese or Korean populations. Notwithstanding the interesting findings, there were concerns about these studies. First, only one of them controlled for ALT and GGT^23^, both of which have been associated with liver fat accumulation^25^ Second, most of them used a small sample size and/or a few incident cases, whereas those with a large sample size comprised primarily men^22, 23^.

Alkaline phosphatase (ALP), an enzyme presented primarily in bone and liver, has been found to elevate in diabetes patients compared with non-diabetes control group^26^. However, other studies found no significant association between ALP and incident diabetes^5, 8, 27, 28^.

The aims of the study were: (1) to examine whether baseline NAFLD status and ALT, AST, GGT, and ALP levels are independent risk factors for incident diabetes, and (2) to determine whether these associations vary by gender group. The study population comprised 132,377 non-type 2 nor type 1 diabetic individuals (64,875 men and 67,502 women) aged 35–79 years. They had first physical examination on 1 January 1996 or later and had one or more follow-up examinations on or before 31 December 2014. To our knowledge, this is the largest and the first prospective study that includes five liver-related factors and evaluates their individual effects on incident diabetes, adjusting for a cluster of classical risk factors.

## Results

Among 132,377 nondiabetic individuals aged 35–79 at baseline, 6,555 (3,734 men and 2,821 women) had a diagnosis of diabetes after the initial examination. The diagnosis of diabetes was made after the entry time by an average of 6.2 (range, 1 to 18.8) years and 6.5 (range, 1 to 18.5) years, respectively, in men and women. Incidence rate of diabetes was 9.97 (95% CI 9.65–10.29) per 1000 person-years among men and 7.11 (6.84–7.37) per 1000 person-years for women. After adjusting for age, the rate was 7.55 (95% CI 7.31–7.80) and 5.44 (5.24–5.65) per 1000 person-years in men and women, respectively. Our numbers are similar to those of the general population reported in Jiang *et al*.^29^ (7.8–8.7 per thousand) and Lin *et al*.^30^ (7.7–9.0 per thousand), suggesting that the study population is comparable to the general population.

Baseline characteristics in individuals with and without incident diabetes were shown in Table 1. Those who developed diabetes were more likely to have NAFLD and elevated levels of ALT, AST, GGT and ALP at baseline (p < 0.01). Additionally, they were older and more likely to have hypertension and a family history of diabetes. They also tend to have elevated levels of BMI, FPG, triglycerides and total cholesterol and a reduced level of high-density lipoprotein cholesterol (HDL-C) (p < 0.01). For diabetic men, they were more likely to be smokers and drinkers but less likely to have active physical activity(p < 0.01).

**Table 1:** Baseline characteristics of the participants with and without incident type 2 diabetes.

	Men (n = 64875)		Women (n = 67502)	
	Non-diabetes (n = 61141)	Diabetes (n = 3734)	Non-diabetes (n = 64681)	Diabetes (n = 2821)
***Demographic Variables***				
Age (years)	44.5 ± 10.1	47.9 ± 10.6	44.9 ± 9.8	51.9 ± 10.0
Family history of diabetes %, (n)	21.9 (13405)	31.0 (1157)	23.9 (15477)	28.9 (814)
Diagnosis of hypertension %, (n)	17.3 (10580)	30.9 (1154)	13.1 (8453)	37.4 (1054)
BMI (kg/m^2^)	24.0 ± 3.0	26.0 ± 3.2	22.5 ± 3.3	25.6 ± 3.7
***Health Behavior***				
Smoking, yes %, (n)	36.2 (22114)	40.5 (1513)	4.8 (3073)	3.6 (101)
Drinking, yes %, (n)	29.2 (17834)	35.0 (1305)	5.9 (3789)	6.6 (187)
Physical activity, active^a^ %, (n)	55.7 (34059)	53.8 (2008)	47.7 (30850)	49.2 (1387)
***Clinical***				
FPG (mg/dL)	97.7 ± 7.8	107.5 ± 9.8	94.5 ± 7.8	105.9 ± 10.3
Total cholesterol (mg/dL)	199.9 ± 34.8	207.3 ± 37.1	195.4 ± 35.7	211.0 ± 39.0
Triglycerides (mg/dL)^b^				
1996–2004	132.8 ± 76.0	172.5 ± 98.6	99.5 ± 56.7	148.9 ± 92.2
2005–2014	132.0 ± 76.3	175.6 ± 92.9	87.6 ± 50.8	139.0 ± 68.9
HDL-C (mg/dL)	47.6 ± 12.7	43.0 ± 11.9	59.0 ± 15.5	50.7 ± 14.3
Diagnosis of NAFLD %, (n)	41.1 (25147)	61.4 (2294)	20.9 (13528)	51.1 (1441)
ALT (U/L)	33.4 ± 35.3	43.5 ± 33.7	20.7 ± 20.5	31.1 ± 33.3
AST (U/L)	25.4 ± 18.1	29.2 ± 17.0	21.3 ± 13.1	26.5 ± 22.0
GGT (U/L)	31.5 ± 34.7	44.6 ± 50.1	18.0 ± 20.7	27.2 ± 27.7
ALP (U/L)^b^				
1996–2004	144.1 ± 43.3	149.5 ± 44.7	128.1 ± 49.4	151.7 ± 54.7
2005–2014	66.2 ± 16.9	69.3 ± 19.1	57.6 ± 19.1	68.8 ± 19.7

The values were described as mean ± SD for a continuous variable, and as frequency (n) for a categorical variable. Abbreviations: FPG, fasting plasma glucose; HDL-C, high-density lipoprotein cholesterol; NAFLD, non alcoholic fatty acid disease; ALT, alanine transaminase; AST, aspartate transaminase; GGT, gamma-glutamyltransferase; ALP, alkaline phosphatase.

^a^Participants with active physical activity: metabolic equivalent of task (MET)-hours per week ≥3.75. ^b^Because a new analyzer was used in 2005 (using a different analysis method), the reference rage of Triglycerides and ALP are different before and after 2005.

### Prediction Power of Risk Factors

We used Cox proportional hazard models to examine the prediction power of each factor for incident diabetes in men (Table 2) and women (Table 3) independently. We tested the proportional hazard assumption using the Schoenfeld and scaled Schoenfeld residuals. Because the proportional hazard assumption was not satisfied for FPG and FPG was not measured periodically, we excluded FPG from the models in both men and women. In addition, the proportional hazard assumption was not satisfied for physical activity in men; we decided to stratify the model of men by physical activity. We further used variance inflation factor (VIF) to test whether multicollinearity existed in our models. VIFs of these factors are <3 (Supplemental Table 3), suggesting no sign of multicollinearity. Using multiple cox regression models, we found that NAFLD, ALT, AST, GGT and ALP were significantly independent risk factors for incident diabetes in both sexes; adjusted hazard ratio (HR) and 95% confidence interval (CI) was 2.08 (1.93–2.23), 1.27 (1.16–1.38), 1.23 (1.13–1.34), 1.58 (1.46–1.72) and 1.37 (1.17–1.60), respectively, in men and was 2.65 (2.43–2.88), 1.56 (1.37–1.77), 1.18 (1.04–1.34), 1.48 (1.32–1.65) and 1.44 (1.25–1.66), respectively, in women. The associations of classical risk factors with diabetes were consistent with previous studies, except that the HRs of smoking and physical activity were not statistically significant in women (p > 0.05).

**Table 2:** Cox regression analysis of type 2 diabetes in men.

	Non-diabetes (n = 61141)	Diabetes (n = 3734)	Crude		Adjusted^c^	
			HR	95% CI	HR	95% CI
***Demographic Variables***						
Ag ≥65 years	5.8 (3560)	8.4 (314)	1.94^***^	[1.73,2.18]	2.19^***^	[1.94,2.47]
Family history of diabetes (yes)	21.9 (13405)	31.0 (1157)	1.69^***^	[1.57,1.81]	1.59^***^	[1.48,1.70]
Hypertension (yes)	17.3 (10580)	30.9 (1154)	2.56^***^	[2.38,2.74]	1.83^***^	[1.70,1.97]
BMI ≥27 kg/m^2^	14.7 (8977)	32.8 (1224)	3.24^***^	[3.03,3.47]	1.82^***^	[1.69,1.96]
***Health Behavior***						
Smoking (yes)	36.2 (22114)	40.5 (1513)	1.23^***^	[1.15,1.31]	1.18^***^	[1.10,1.27]
Drinking (yes)	29.2 (17834)	35.0 (1305)	1.16^***^	[1.08,1.24]	1.08^*^	[1.00,1.16]
Physical activity (active^a^)	55.7 (34059)	53.8 (2008)	0.85^***^	[0.80,0.91]	NA^c^	
***Clinical***						
Total cholesterol >240 mg/dL	12.0 (7364)	17.2 (642)	1.49^***^	[1.37,1.63]	1.18^***^	[1.08,1.28]
TG >200 or >150 mg/dL^b^	9.3 (5672)	22.7 (846)	2.28^***^	[2.12,2.47]	1.33^***^	[1.23,1.45]
HDL-C ≥40 mg/dL	73.0 (44613)	59.5 (2184)	0.66^***^	[0.62,0.71]	0.81^***^	[0.75,0.87]
NAFLD (yes)	41.1 (25147)	61.4 (2294)	3.09^***^	[2.89,3.30]	2.08^***^	[1.93,2.23]
ALT >33 U/L	32.2 (19704)	49.8 (1858)	2.31^***^	[2.16,2.46]	1.27^***^	[1.16,1.38]
AST >27 U/L	24.5 (15005)	38.2 (1428)	2.12^***^	[1.99,2.27]	1.23^***^	[1.13,1.34]
GGT ≥50 U/L	12.7 (7735)	24.8 (927)	2.70^***^	[2.50,2.90]	1.58^***^	[1.46,1.72]
ALP >220 or >104 U/L^b^	2.7 (1621)	4.6 (171)	1.60^***^	[1.37,1.86]	1.37^***^	[1.17,1.60]

The values were described as frequency (n). ^*^ *p* < 0.05, ^**^ *p* < 0.01, ^***^ *p* < 0.001. 95% CI in brackets. Abbreviations: FPG, fasting plasma glucose; TG, triglycerides; HDL-C, high-density lipoprotein cholesterol; NAFLD, non alcoholic fatty acid disease; ALT, alanine transaminase; AST, aspartate transaminase; GGT, gamma-glutamyltransferase; ALP, alkaline phosphatase. ^a^Active: metabolic equivalent of task (MET)-hours per week ≥3.75. ^b^Because a new analyzer was used in 2005, the abnormal range for TG is >200 mg/dL (2000–2004) or >150 mg/dL (2005-); for ALP is >220 U/L (2000–2004) or >104 U/L (2005-). ^c^Multiple cox regression. Because the proportional hazard assumption was not satisfied for physical activity, the model was stratified by physical activity.

**Table 3:** Cox regression analysis of type 2 diabetes in women.

	Non-diabetes (n = 64681)	Diabetes (n = 2821)	Crude		Adjusted^c^	
			HR	95% CI	HR	95% CI
***Demographic Variables***	% (n)	% (n)				
Age ≥65 years	4.4 (2843)	10.3 (291)	3.62^***^	[3.20,4.09]	1.73^***^	[1.52,1.97]
Family history of diabetes (yes)	23.9 (15477)	28.9 (814)	1.45^***^	[1.33,1.57]	1.59^***^	[1.47,1.73]
Hypertension (yes)	13.1 (8453)	37.4 (1054)	4.50^***^	[4.17,4.86]	2.25^***^	[2.07,2.45]
BMI ≥27 kg/m^2^	9.0 (5822)	31.8 (896)	5.17^***^	[4.78,5.60]	2.04^***^	[1.87,2.23]
***Health Behavior***						
Smoking (yes)	4.8 (3073)	3.6 (101)	0.87	[0.71,1.06]	0.95	[0.78,1.17]
Drinking (yes)	5.9 (3789)	6.6 (187)	1.10	[0.95,1.28]	1.32^***^	[1.13,1.53]
Physical activity (active^a^)	47.7 (30850)	49.2 (1387)	1.00	[0.93,1.08]	0.98	[0.91,1.05]
***Clinical***						
Total cholesterol >240 mg/dL	10.6 (6829)	21.0 (593)	2.33^***^	[2.13,2.56]	1.47^***^	[1.34,1.62]
TG >200 or >150 mg/dL^b^	3.6 (2307)	15.9 (448)	4.27^***^	[3.86,4.72]	1.50^***^	[1.34,1.67]
HDL-C ≥50 mg/dL	71.5 (46236)	48.8 (1377)	0.48^***^	[0.44,0.51]	0.66^***^	[0.61,0.71]
NAFLD (yes)	20.9 (13528)	51.1 (1441)	5.25^***^	[4.87,5.66]	2.65^***^	[2.43,2.88]
ALT >33 U/L	9.2 (5957)	25.4 (716)	3.66^***^	[3.36,3.98]	1.56^***^	[1.37,1.77]
AST >27 U/L	11.1 (7166)	25.2 (711)	3.03^***^	[2.78,3.29]	1.18^**^	[1.04,1.34]
GGT ≥39 U/L	5.8 (3763)	16.1 (455)	3.47^***^	[3.14,3.84]	1.48^***^	[1.32,1.65]
ALP >220 or >104 U/L^b^	2.5 (1591)	7.7 (218)	3.22^***^	[2.81,3.70]	1.44^***^	[1.25,1.66]

The values were described as frequency (n). ^*^ *p* < 0.05, ^**^ *p* < 0.01, ^***^ *p* < 0.001. 95% CI in brackets. Abbreviations: FPG, fasting plasma glucose; TG, triglycerides; HDL-C, high-density lipoprotein cholesterol; NAFLD, non alcoholic fatty acid disease; ALT, alanine transaminase; AST, aspartate transaminase; GGT, gamma-glutamyltransferase; ALP, alkaline phosphatase. ^a^Active: metabolic equivalent of task (MET)-hours per week ≥3.75. ^b^Because a new analyzer was used in 2005, the abnormal range for TG is >200 mg/dL (2000–2004) or >150 mg/dL (2005-); for ALP is >220 U/L (2000–2004) or >104 U/L (2005-). ^c^Multiple cox regression model.

We further made stratifications for liver-related enzymes to investigate whether individuals with extreme high level of liver-related enzymes are associated with high risk of diabetes. We classified participants with abnormal level of liver enzymes into two groups, high and extreme high. Participants classified as extreme high were those whose enzyme level were at top 25% of the abnormal values. We observed mild increases in HRs for extreme high level of liver-related enzymes in men (Table 4). However, in women an increase in HR was observed only for AST, but not for ALT, GGT and ALP (Table 5). HR was not significant for AST level between 27 and 40 U/L (HR = 1.11, CI = 0.97–1.27). Only those with AST level >40 U/L had an increased risk of diabetes (HR = 1.58, CI = 1.27–1.96). Our stratification suggests AST cut-point for women should be 40 U/L, not the one currently used in clinical check-ups (27 U/L).

**Table 4:** Adjusted hazard ratio (HR) of type 2 diabetes in men (stratification for liver-related enzymes).

	Non-diabetes (n = 61141)	Diabetes (n = 3734)	Adjusted^c^	
			HR	95% CI
***Demographic Variables***	% (n)	% (n)		
Age ≥65 years	5.8 (3560)	8.4 (314)	2.21^***^	[1.95,2.49]
Family history of diabetes (yes)	21.9 (13405)	31.0 (1157)	1.58^***^	[1.47,1.70]
Hypertension (yes)	17.3 (10580)	30.9 (1154)	1.83^***^	[1.70,1.97]
BMI ≥27 kg/m^2^	14.7 (8977)	32.8 (1224)	1.80^***^	[1.67,1.94]
***Health Behavior***				
Smoking (yes)	36.2 (22114)	40.5 (1513)	1.18^***^	[1.10,1.27]
Drinking (yes)	29.2 (17834)	35.0 (1305)	1.09^*^	[1.02,1.17]
Physical activity (active^a^)	55.7 (34059)	53.8 (2008)	NA^c^	
***Clinical***				
Total cholesterol >240 mg/dL	12.0 (7364)	17.2 (642)	1.18^***^	[1.08,1.28]
TG >200 or >150 mg/dL^b^	9.3 (5672)	22.7 (846)	1.34^***^	[1.23,1.45]
HDL-C ≥40 mg/dL	73.0 (44613)	59.5 (2184)	0.81^***^	[0.75,0.87]
NAFLD (yes)	41.1 (25147)	61.4 (2294)	2.08^***^	[1.93,2.24]
ALT (U/L)				
(33–63]	24.8 (15160)	33.2 (1238)	1.24^***^	[1.14,1.35]
(63 –]	7.4 (4544)	16.6 (620)	1.44^***^	[1.24,1.67]
AST (U/L)				
(27–40]	18.8 (11493)	25.3 (943)	1.12^*^	[1.02,1.23]
(40 –]	5.7 (3512)	13.0 (485)	1.36^***^	[1.17,1.58]
GGT (U/L)				
[50–100)	9.7 (5935)	18.6 (696)	1.49^***^	[1.35,1.63]
[100 –]	2.9 (1800)	6.2 (231)	1.59^***^	[1.37,1.84]
ALP (U/L)				
(220–265] or (104–125]^b^	2.8 (1714)	4.1 (153)	1.30^**^	[1.11,1.54]
(265 –] or (125 –]^b^	0.8 (459)	1.2 (52)	1.54^**^	[1.16,2.03]

95% confidence intervals (CI) in brackets. ^*^ *p* < 0.05, ^**^ *p* < 0.01, ^***^ *p* < 0.001.

Abbreviations: FPG, fasting plasma glucose; TG, triglycerides; HDL-C, high-density lipoprotein cholesterol; NAFLD, non alcoholic fatty acid disease; ALT, alanine transaminase; AST, aspartate transaminase; GGT, gamma-glutamyltransferase; ALP, alkaline phosphatase.

^a^Active: metabolic equivalent of task (MET)-hours per week ≥3.75. ^b^Because a new analyzer was used in 2005, the abnormal range for TG is >200 mg/dL (2000–2004) or >150 mg/dL (2005-); the cut-points for high ALP are >220 (2000–2004) or >104 (2005-) and for extreme high level are >265 U/L (2000–2004) or >125 U/L (2005-). ^c^Because the proportional hazard assumption was not satisfied for physical activity, the multiple cox regression model was stratified by physical activity.

**Table 5:** Adjusted hazard ratio (HR) of type 2 diabetes in women (stratification for liver-related enzymes).

	Non-diabetes (n = 64681)	Diabetes (n = 2821)	Adjusted^c^	
			HR	95% CI
***Demographic Variables***	% (n)	% (n)		
Age ≥65 years	4.4 (2843)	10.3 (291)	1.70^***^	[1.49,1.94]
Family history of diabetes (yes)	23.9 (15477)	28.9 (814)	1.59^***^	[1.46,1.73]
Hypertension (yes)	13.1 (8453)	37.4 (1054)	2.25^***^	[2.07,2.45]
BMI ≥27 kg/m^2^	9.0 (5822)	31.8 (896)	2.03^***^	[1.86,2.22]
***Health Behavior***				
Smoking (yes)	4.8 (3073)	3.6 (101)	0.95	[0.77,1.16]
Drinking (yes)	5.9 (3789)	6.6 (187)	1.32^***^	[1.14,1.54]
Physical activity (active^a^)	47.7 (30850)	49.2 (1387)	0.98	[0.91,1.05]
***Clinical***				
Total cholesterol >240 mg/dL	10.6 (6829)	21.0 (593)	1.49^***^	[1.36,1.64]
TG >200 or >150 mg/dL^b^	3.6 (2307)	15.9 (448)	1.50^***^	[1.34,1.67]
HDL-C ≥50 mg/dL	71.5 (46236)	48.8 (1377)	0.66^***^	[0.61,0.71]
NAFLD (yes)	20.9 (13528)	51.1 (1441)	2.65^***^	[2.44,2.88]
ALT (U/L)				
(33–63]	7.1 (4620)	17.9 (506)	1.49^***^	[1.30,1.70]
(63 –]	2.1 (1337)	7.4 (210)	1.37^*^	[1.07,1.74]
AST (U/L)				
(27–40]	8.3 (5349)	15.1 (426)	1.11	[0.97,1.27]
(40 –]	2.8 (1817)	10.1 (285)	1.58^***^	[1.27,1.96]
GGT (U/L)				
[39–78)	4.6 (2957)	12.3 (348)	1.46^***^	[1.29,1.66]
[78 –]	1.3 (806)	3.8 (107)	1.39^**^	[1.13,1.72]
ALP (U/L)				
(220–265] or (104–125]^b^	2.3 (1472)	5.6 (158)	1.44^***^	[1.23,1.70]
(265 –] or (125 –]^b^	0.9 (605)	2.9 (81)	1.25	[0.99,1.57]

95% confidence intervals (CI) in brackets. ^*^ *p* < 0.05, ^**^ *p* < 0.01, ^***^ *p* < 0.001.

Abbreviations: FPG, fasting plasma glucose; TG, triglycerides; HDL-C, high-density lipoprotein cholesterol; NAFLD, non alcoholic fatty acid disease; ALT, alanine transaminase; AST, aspartate transaminase; GGT, gamma-glutamyltransferase; ALP, alkaline phosphatase.

^a^Active: metabolic equivalent of task (MET)-hours per week ≥3.75. ^b^Because a new analyzer was used in 2005, the abnormal range for TG is >200 mg/dL (2000–2004) or >150 mg/dL (2005-); the cut-points for high ALP are >220 (2000–2004) or >104 (2005-) and for extreme high level are >265 U/L (2000–2004) or >125 U/L (2005-). ^c^Multiple cox regression model.

### Sensitivity Analysis

We further excluded individuals with viral hepatitis B or whose liver fat was likely caused by excessive alcohol consumption: positive for hepatitis B virus surface antigen (n = 18530), GGT level (a common marker of alcohol consumption) >100 U/L (n = 1034 men) or 78 U/L (n = 368 women), AST/ALT concentrations >2 (n = 191), or heavy drinker (men > 140 g ethanol /per week or 50 g ethanol /each time; women >70 g ethanol/per week or 40 g ethanol /each time) (n = 3878) according to the suggestion of National Institutes of Health Clinical Research Network^31^. The associations were virtually the same (Table 6 and Supplemental Table 4).

**Table 6:** Adjusted hazard ratio (HR) of type 2 diabetes in men and women excluding individuals with excessive alcohol consumption or viral hepatitis B (stratification for liver-related enzymes).

	Men (n = 49635)		Women (n = 58751)	
	HR	95% CI	HR	95% CI
***Demographic Variables***				
Age ≥65 years	2.23^***^	[1.95,2.56]	1.76^***^	[1.53,2.02]
Family history of diabetes (yes)	1.65^***^	[1.51,1.79]	1.59^***^	[1.46,1.74]
Hypertension (yes)	1.83^***^	[1.68,2.00]	2.21^***^	[2.01,2.42]
BMI (≥27)	1.97^***^	[1.80,2.15]	2.07^***^	[1.88,2.28]
***Health Behavior***				
Smoking (yes)	1.21^***^	[1.12,1.32]	0.89	[0.71,1.13]
Drinking (yes)	1.06	[0.96,1.17]	1.42^***^	[1.19,1.70]
Physical activity (active^a^)	NA^e^		1.00	[0.92,1.08]
***Clinical***				
Total cholesterol >240 mg/dL	1.17^**^	[1.06,1.30]	1.53^***^	[1.38,1.69]
TG >200 or >150 mg/dL^b^	1.36^***^	[1.24,1.51]	1.50^***^	[1.33,1.68]
HDL-C ≥40 or ≥50 mg/dL^c^	0.81^***^	[0.75,0.88]	0.67^***^	[0.61,0.73]
NAFLD (yes)	1.98^***^	[1.81,2.16]	2.70^***^	[2.46,2.96]
ALT (U/L)				
(33–63]	1.21^***^	[1.10,1.35]	1.52^***^	[1.31,1.77]
(63 –]	1.44^***^	[1.21,1.72]	1.38^*^	[1.05,1.81]
AST (U/L)				
(27–40]	1.16^*^	[1.03,1.30]	1.09	[0.94,1.26]
(40 –]	1.40^***^	[1.16,1.69]	1.57^***^	[1.22,2.01]
GGT (U/L)				
[39–78) or [50–100)^d^	1.41^***^	[1.25,1.60]	1.63^***^	[1.41,1.88]
[78 –] or [100 –]^d^	1.55^***^	[1.31,1.82]	1.27^*^	[1.04,1.56]
ALP (U/L)				
(220–265] or (104–125]^b^	1.39^***^	[1.15,1.68]	1.42^***^	[1.18,1.70]
(265 –] or (125 –]^b^	1.30	[0.90,1.86]	1.42^**^	[1.10,1.85]

95% confidence intervals (CI) in brackets. ^*^ *p* < 0.05, ^**^ *p* < 0.01, ^***^ *p* < 0.001.

Abbreviations: FPG, fasting plasma glucose; TG, triglycerides; HDL-C, high-density lipoprotein cholesterol; NAFLD, non alcoholic fatty acid disease; ALT, alanine transaminase; AST, aspartate transaminase; GGT, gamma-glutamyltransferase; ALP, alkaline phosphatase.

^a^Active: metabolic equivalent of task (MET)-hours per week ≥3.75. ^b^Because a new analyzer was used in 2005, the abnormal range for TG is >200 mg/dL (2000–2004) or >150 mg/dL (2005-); the cut-points for high ALP are >220 (2000–2004) or >104 (2005-) and for extreme high level are >265 U/L (2000–2004) or >125 U/L (2005-). ^c^The cut-points for high level of HDL-C are different in men (≥40 mg/dL) and women (≥50 mg/dL). ^d^The cut-points for high and extreme high levels of GGT are different in men and women; for men the cut-points are >50 and >100 U/L, respectively, and for women the cut-points are >39 and >78, respectively. ^e^The model was stratified by physical activity because the proportional hazard assumption was not satisfied for physical activity in men.

## Discussion

Based on a large sample size of over 132,000 adults without diabetes at baseline, we found that NAFLD, ALT, AST, GGT and ALP are independent predictors of incident diabetes in both men and women. Liver fat has been associated with several features of insulin resistance^16^. Our finding is consistent with previous studies^4, 18–24^ by demonstrating that ultrasonography-diagnosed NAFLD is a risk factor for incident diabetes. Nonetheless, to assess the role of NAFLD in incident diabetes, previous studies included only ALT^18–20, 22^ or used male sample for study when ALT, AST and GGT were included^23^. The present study advanced the previous studies by confirming that NAFLD is an independent risk factor for incident diabetes by taking ALT, AST, GGT and ALP all into account using a large sample including both men and women.

The prevalence of NAFLD in non-diabetic men was significantly higher than that in non-diabetic women (41% in men vs. 21% in women) in our cohort, which is consistent with the finding in a study of the US adult men and women^32^. The larger BMI value of non-diabetic men compared to non-diabetic women in our cohort might account for some of the difference, because obesity has been reported as a risk factor for NAFLD^33^. Additionally, the prevalence of NAFLD in non-diabetic men in our cohort was higher than that in other studies (41% vs. 27–34%)^19, 22^. Possible reasons for the difference include ethnicity, different proportion of overweight/obesity individuals included and/or various acceptable levels of alcohol intake to define NAFLD.

Patients with NAFLD have been characterized by elevated ALT, AST and GGT^34^. Therefore, these hepatic enzymes were used as markers for liver fat accumulation to predict incident diabetes before direct measurements of liver fat became easily available^6–8^. However, prediction powers of ALT, AST and GGT for incident diabetes have been inconclusive. The present study found independent effects of ALT, AST and GGT, but raised the question that why these hepatic enzymes still predicted incident diabetes after controlling for direct measurement of liver fat (i.e., ultrasonography-diagnosed NAFLD). It has been reported that ultrasonography fails to detect minor fat accumulation (fatty infiltration <30%)^35^ or the presence of NAFLD in patients with chronic hepatitis C^36^. Therefore, elevated levels of ALT, AST and GGT may give insights into liver fat content in people whose liver fat cannot be correctly diagnosed by ultrasonography. In addition, the prevalence of diabetes was reported high in patients with liver diseases, including cirrhosis^37^ and hepatitis C^38^, who tend to have elevated levels of hepatic enzymes. The independent effects of ALT, AST, and GGT found in the present study are in line with the role of liver diseases and liver injury in the development of diabetes.

ALP elevation has been commonly seen in patients with bones diseases and/or renal hyperfiltration^39^, and bone diseases and renal hyperfiltration have been associated with diabetic patients^40^. In particular, renal hyperfiltration has been observed in patients with newly diagnosed type 2 diabetes^40^. However, many studies reported no association between ALP level and diabetes risk^5, 8, 27, 28, 41^. The present study found ALP an independent risk factor for incident diabetes after controlling other liver-related factors. However, further investigations on the causal effects of ALP level on the development of diabetes are needed.

Our results offer potential implications for clinical practice. Since the measurements of ultrasonography-diagnosed NAFLD, ALT, AST, GGT, and ALP are well standardized and available in routine clinical practice, these markers can be included in future diabetes prediction algorithms. Targeted approaches to control the level of each risk factor may considerably reduce the risk of diabetes.

The current study is notable for having several important strengths, including large sample size and large number of incident cases, comparison of the five liver-related factors that are available in clinical practice, and inclusion of full classical confounders. Nonetheless, our study has some limitations. First, we used ultrasonography, the most commonly used and reasonably accurate method to diagnose NAFLD. However, ultrasonography is affected by body mass and cannot detect minor liver fat accumulation (fatty infiltration <30%) or accurately quantify liver fat. Moreover, ultrasonography has low sensitivity in detecting liver fat on biopsy in patients with chronic hepatitis C^36^. Second, we did not have information on HbA1C, a more accurate glucose measure to define diabetic patients, or baseline insulin resistance, which has been shown to associated with incident type 2 diabetes. Third, it is not clear that elevated ALP results from liver disease, bone disease or both; a test for ALP isoenzymes may be necessary to determine the cause. Forth, we only had one measure for each enzyme at baseline, but ALT and AST have been found to have high short-term variability^42^.

In summary, we report that NAFLD, ALT, AST, GGT and ALP are independent risk factors for incident diabetes in both men and women, resolving the controversy between previous studies. Our results highlight complex pathophysiological relationships between diabetes, bone diseases and kidney function. We suggest that ALP should be considered in future algorithms to predict diabetes.

## Research Design and Methods

### Study design and participants

The study protocol was approved by the Institutional Review Board of Taipei Medical University and the methods were carried out in accordance with the relevant guidelines and regulations. Data used in this study were extracted from the MJ Health Database, a large-scale health research database derived from an ongoing physical examination service in Taiwan, the MJ Health Management Institution^43^. The participants were generally healthy people who participated in two or more health examinations between 1 January 1996 and 31 December 2014. Only those participants with signed informed consent were included in the MJ Health Database and personal identifiers were removed when data release for the purpose of research. The health evaluation in initial and follow-up examinations was performed using same protocol, including anthropometric, blood pressure, and biochemical measurements, and a complete physical examination. All participants were instructed to complete a self-administered questionnaire before the time of screening to gather information on demographic, personal and family history of major chronic diseases and lifestyle (e.g., smoking, alcohol consumption and physical activity). Weight and height measurements were made at the clinic using KN-5000 A auto anthropometer (Nakamura, Tokyo) on barefoot subjects wearing light indoor clothing. They were also required to fast for at least 8-hour before arriving at the clinic. Fasting blood were collected and analyzed by Hitachi 7150 (1996–2004) and TOSHIBA C8000 (2005-) auto-analyzers for blood glucose, a panel of lipid profile, liver profile (including ALT, AST, and GGT) and ALP^43^. The two machines used different analysis methods for triglycerides and ALP and generate different reference values (Supplemental Table 1). So, the cut-points for high level of triglycerides and ALP are different after 2005. The reference value for triglycerides and ALP before 2005 is 35–200 mg/dl and 66–220 U/L, respectively, whereas the value from 2005 is 0–150 mg/dl and 35–104 U/L, respectively. The mean and standard deviation of these tests in two machines were in Supplemental Table 2. A complete physical examination was performed by a family physician.

For each individual, the entry date to this study was the date of first health examination, while the exit date (right censor date) was the first onset of diabetes in the follow-up examination or date of the last examination. We initially identified 175,525 adults aged 35–79 years at the entry date. We excluded individuals with type 2 diabetes at baseline (n = 9,747) or missing information on one or more risk factors under study (n = 25,678). Individuals who stayed in the study less than 365 days were excluded (n = 6,948). Individuals taking glucose-lowering medication without information on self-reported diabetes were also excluded (n = 775). The final population for this study included 132,377 non-diabetic individuals (64,875 men and 67,502 women) with an average follow-up of 5.8 years (range, 1 to 18.9) for men and 5.9 (1 to 18.9) for women, and thus contributed 771,489 person years of observations (374,465 in men and 397,024 in women).

### Assessment of diabetes

The primary outcome was the first-onset of diabetes in the follow-up examination, i.e., incident diabetes. Diabetes was defined as individuals who had fasting plasma glucose (FPG) ≥ 126 mg/dL (6.99 mmol/L) (test-based diabetes, n = 6,138) or self-reported diabetic patients who currently take glucose-lowering medication (known diabetes, n = 417).

### Diabetic risk factors

Baseline biometric and risk factor data were derived from the first physical examination and health questionnaire. NALFD status was ultrasonography-diagnosed by well-trained clinicians to be no (negative) or yes (positive). The level of each liver enzyme was categorized as high (ALT >33 U/L; AST >27 U/L; GGT ≥50 U/L [men] or ≥39 U/L [women]; ALP >220 U/L [1996–2004] or >104 U/L [2005-present]) and normal (otherwise). Age status was categorized as old (≥65 years) and young (otherwise). Hypertension was defined as systolic blood pressure ≥140 mmHg and/or diastolic blood pressure ≥90 mmHg and/or on anti-hypertensive drugs. Obesity was defined as a body mass index (BMI) ≥27 kg/m^2^, which was calculated as weight (in kilogram) divided by the square of height (in meter). Smoking status was categorized as current smoking and not current smoking (never or former). Drinking status was categorized as drinking (>20 g ethanol/week, calculated by the type of alcohol and the number of drinks a week) and not drinking. Physical activity was determined by metabolic equivalent of task (MET)-hours per week and categorized as active (≥3.75 MET-h) and inactive (otherwise)^44^. The levels of triglycerides and total cholesterol were also categorized as high (triglycerides >200 mg/dL [1996–2004] or >150 mg/dL [2005-present]; total cholesterol ≥240 mg/dL) and normal (otherwise). The level of high-density lipoprotein cholesterol (HDL-C) was categorized as normal (≥40 mg/dL [men] or ≥50 mg/dL [women]) and low (otherwise).

### Statistical analysis

Descriptive data were presented as frequency (the number of participants) and mean ± SD, respectively, for categorical and continuous variables. We used Cox proportional hazard models to examine the effect of individual factor on diabetes. For multiple regression analysis, the relationships of diabetes with NAFLD (yes, no), ALT (high, normal), AST (high, normal), GGT (high, normal), and ALP (high, normal) were assessed by controlling for other risk factors, including age (old, young), family history of diabetes (yes, no), hypertension (yes, no), BMI (obese, non-obese), smoking (yes, no), drinking (yes, no), physical activity (active, inactive), triglyceride (high, normal), total cholesterol (high, normal), and HDL-C (normal, low). The analyses were conducted separately for men and women. Hazard ratios were considered statistically significant if their 95% CI did not include 1.0. All statistical analyses were carried out using Stata version 13.1 (Stata, College Station, Texas).

## Electronic supplementary material

Supplemental Tables
