# Supplementary material for: Liver Fat, Hepatic Enzymes, Alkaline Phosphatase and the Risk of Incident Type 2 Diabetes: A Prospective Study of 132,377 Adults
Source: Sci Rep. 2017 Jul 5;7:4649. doi: 10.1038/s41598-017-04631-7 (PMC5498613; doi:10.1038/s41598-017-04631-7)
Supplement: Supplementary file 1 — Supplemental Tables [file 41598_2017_4631_MOESM1_ESM.pdf]

TITLE PAGE

Title: Liver Fat, Hepatic Enzymes, Alkaline Phosphatase and the Risk of Incident Type 2 Diabetes: A Prospective Study of 132,377 Adults

Short running title: Liver-Related Risk Factors for Type 2 Diabetes

Authors

Sean Chun-Chang Chen [1]

Shan Pou Tsai [2]

Wun-Kai Jiang [2]

Jing-Yun Jhao [2]

Chwen Keng Tsao [2]

Ly-Yun Chang [2, 3*]

Affiliations

[1]*Graduate Institute of Biomedical Informatics, College of Medical Science and Technology, Taipei Medical University, Taipei, 110, Taiwan*

[2]*MJ Health Management Institution, Taipei, 114, Taiwan.*

[3]*Institute of Sociology, Academia Sinica, Taipei, 115, Taiwan.*

[*]**Corresponding author:** Ly-Yun Chang; phone: +886-2-2793-7878 #22; fax: +886-2-2793-0399; email: sociochang@gmail.com

Supplemental Table 1 Analysis methods and reference value of two different machines, HITACHI 7150 (1995-2004) and TOSHIBA C8000 (2005-).

	HITACHI 7150 (1995-2004)			TOSHIBA C8000 (2005-)		
	Analysis Method	Reference	SE	Analysis Method	Reference	SE
**TG**	Free Glycerol Blanking Method	35-200 mg/dl	NA[a]	GPO-POD-ESPT	≦150mg/dl	1.0-1.6
**ALT**	UV with P5P	5-33 U/L	NA[a]	UV with P5P	5-33 U/L	0.39-1.29
**AST**	UV with P5P	10-27 U/L	NA[a]	UV with P5P	10-27 U/L	0.65-1.43
**GGT**	γ-L-Glutamyl-p-nitroanilide Chlorate Substrate	M: <50 U/L<br>W: <39 U/L	NA[a]	Glu-3-CA-4NA	M:＜50 U/L<br>W:＜39 U/L	0.45-1.24
**ALP**	Kinetic assay	66-220 U/L	NA[a]	PNPPDEA (diethanolamine)	35-104 U/L	5.9-8.3

Abbreviations: SE: standard error, TG, triglycerides; ALT, alanine transaminase; AST, aspartate transaminase; GGT, gamma-glutamyltransferase; ALP, alkaline phosphatase. M: men; W: women. [a] NA: the vendor was unable to provide SE.

Supplemental Table 2 Mean and SD of clinical tests using two different machines, HITACHI 7150 (1995-2004) and TOSHIBA C8000 (2005-).

	Men (n=65249)		Women (n=67946)	
	Non-diabetes (n=61155)	Diabetes (n=4094)	Non-diabetes (n=64710)	Diabetes (n=3236)
Clinical				
FPG (mg/dL)				
1996-2004	97.1 ± 7.8	106.4 ± 9.8	94.3 ± 7.8	105.2 ± 10.3
2005-2014	98.9 ± 7.6	111.3 ± 10.1	94.8 ± 7.8	110.1 ± 9.0
Total cholesterol (mg/dL)				
1996-2004	200.4 ± 35.2	208.5 ± 37.0	197.0 ± 36.4	211.3 ± 39.2
2005-2014	199.0 ± 34.2	204.2 ± 37.1	192.4 ± 34.1	209.6 ± 38.2
Triglycerides (mg/dL)				
1996-2004	132.8 ± 76.0	172.5 ± 98.6	99.5 ± 56.7	148.9 ± 92.2
2005-2014	132.0 ± 76.3	175.6 ± 92.9	87.6 ± 50.8	139.0 ± 68.9
HDL-C (mg/dL)				
1996-2004	46.0 ± 12.9	42.2 ± 12.2	56.6 ± 15.2	49.9 ± 14.3
2005-2014	50.6 ± 11.6	46.1 ± 10.4	63.5 ± 14.9	54.9 ± 13.5
ALT (U/L)				
1996-2004	31.9 ± 34.5	41.9 ± 33.2	20.6 ± 21.2	30.6 ± 33.9
2005-2014	36.1 ± 36.6	49.3 ± 34.8	20.9 ± 19.2	34.0 ± 30.1
AST (U/L)				
1996-2004	25.3 ± 18.4	28.9 ± 17.4	21.4 ± 13.8	26.4 ± 22.9
2005-2014	25.8 ± 17.6	30.7 ± 15.4	21.1 ± 11.7	27.3 ± 16.6
GGT (U/L)				
1996-2004	29.6 ± 32.7	42.0 ± 48.9	17.4 ± 18.8	26.5 ± 26.5
2005-2014	35.0 ± 37.8	53.9 ± 53.0	18.9 ± 24.0	30.8 ± 33.1
ALP (U/L)				
1996-2004	144.1 ± 43.3	149.5 ± 44.7	128.1 ± 49.4	151.7 ± 54.7
2005-2014	66.2 ± 16.9	69.3 ± 19.1	57.6 ± 19.1	68.8 ± 19.7

Abbreviations: FPG, fasting plasma glucose; HDL-C, high-density lipoprotein cholesterol; ALT, alanine transaminase; AST, aspartate transaminase; GGT, gamma-glutamyltransferase; ALP, alkaline phosphatase.

Supplemental table 3 Variance inflation factors (VIFs) of variables in the models

	VIF	
	Men	Women
Demographic Variables		
Age ≥ 65 (years)	1.12	1.15
Family history of diabetes (yes)	1.26	1.26
Hypertension (yes)	1.31	1.35
BMI (≥27)	1.36	1.30
Health Behavior		
Smoking (yes)	1.55	1.08
Drinking (yes)	1.51	1.10
Physical activity (active[a])	NA[d]	1.65
Clinical		
Cholesterol >240 mg/dL	1.18	1.18
TG >200 or >150 mg/dL[b]	1.19	1.11
HDL-C ≥ 40 or ≥50 mg/dL[c]	2.01	1.86
NAFLD (yes)	1.93	1.48
ALT >33 U/L	2.53	2.10
AST >27 U/L	2.11	2.04
GGT ≥39 or ≥50 U/L[c]	1.39	1.25
ALP >220 or >104 U/L[b]	1.04	1.08
Mean	1.53	1.40

Abbreviations: TG, triglycerides; HDL-C, high-density lipoprotein cholesterol; NAFLD, non alcoholic fatty acid disease; ALT, alanine transaminase; AST, aspartate transaminase; GGT, gamma-glutamyltransferase; ALP, alkaline phosphatase. [a]Active: metabolic equivalent of task (MET)-hours per week ≥ 3.75. [b]Because a new analyzer was used in 2005, the abnormal range for TG is > 200 mg/dL (2000-2004) or >150 mg/dL (2005-); for ALP is >220 U/L (2000-2004) or >104 U/L (2005-). [c] The cut-points for high levels of HDL-C and GGT are different in men (≥ 40 mg/dL for HDL-C and ≥50 U/L for GGT) and women (≥ 50 mg/dL for HDL-C and ≥39 U/L for GGT). [d] The model was stratified by physical activity because the proportional hazard assumption was not satisfied for physical activity in men.

Supplemental Table 4 Adjusted hazard ratio (HR) of type 2 diabetes in men and women excluding individuals with excessive alcohol consumption or viral hepatitis B

	Men (n=49635)		Women (n=58751)	
	HR	95% CI	HR	95% CI
Demographic Variables				
Age ≥ 65 (years)	2.21***	[1.93,2.53]	1.80***	[1.57,2.06]
Family history of diabetes (yes)	1.65***	[1.52,1.79]	1.60***	[1.46,1.75]
Hypertension (yes)	1.84***	[1.68,2.01]	2.21***	[2.02,2.43]
BMI (≥27)	1.99***	[1.82,2.18]	2.07***	[1.88,2.28]
Health Behavior				
Smoking (yes)	1.22***	[1.12,1.32]	0.89	[0.71,1.13]
Drinking (yes)	1.05	[0.95,1.15]	1.41***	[1.18,1.69]
Physical activity (active[a])	NA[d]		1.00	[0.92,1.08]
Clinical				
Total cholesterol >240 mg/dL	1.18**	[1.06,1.30]	1.50***	[1.36,1.66]
TG >200 or >150 mg/dL[b]	1.36***	[1.24,1.51]	1.49***	[1.32,1.67]
HDL-C ≥ 40 or ≥50 mg/dL[c]	0.81***	[0.75,0.88]	0.67***	[0.61,0.73]
NAFLD (yes)	1.98***	[1.81,2.16]	2.71***	[2.47,2.97]
ALT >33 U/L	1.24***	[1.12,1.38]	1.58***	[1.37,1.83]
AST >27 U/L	1.28***	[1.15,1.41]	1.15	[1.00,1.32]
GGT ≥39 or ≥50 U/L [c]	1.52***	[1.37,1.69]	1.53***	[1.35,1.73]
ALP >220 or >104 U/L[b]	1.40***	[1.17,1.69]	1.45***	[1.23,1.70]

95% confidence intervals (CI) in brackets. * $p < 0.05$, ** $p < 0.01$, *** $p < 0.001$.
Abbreviations: TG, triglycerides; HDL-C, high-density lipoprotein cholesterol; NAFLD, non alcoholic fatty acid disease; ALT, alanine transaminase; AST, aspartate transaminase; GGT, gamma-glutamyltransferase; ALP, alkaline phosphatase. [a]Active: metabolic equivalent of task (MET)-hours per week ≥ 3.75. [b] Because a new analyzer was used in 2005, the abnormal range for TG is > 200 mg/dL (2000-2004) or >150 mg/dL (2005-); for ALP is >220 U/L (2000-2004) or >104 U/L (2005-). [c] The cut-points for high levels of HDL-C and GGT are different in men (≥ 40 mg/dL for HDL-C and ≥50 U/L for GGT) and women (≥ 50 mg/dL for HDL-C and ≥39 U/L for GGT). [d] The model was stratified by physical activity because the proportional hazard assumption was not satisfied for physical activity in men.
